# A standardized method for quantifying unidirectional genetic introgression

**DOI:** 10.1002/ece3.1169

**Published:** 2014-07-28

**Authors:** Sten Karlsson, Ola H Diserud, Thomas Moen, Kjetil Hindar

**Affiliations:** 1 Norwegian Institute for Nature Research (NINA)P.O. Box 5685 Sluppen, N-7485, Trondheim, Norway; 2 AquaGen ASP.O. Box 1240, N-7462, Trondheim, Norway; 3 Centre for Integrative Genetics, Norwegian University of Life SciencesArboretveien 6, N-1432, Ås, Norway

**Keywords:** Aquaculture, Atlantic salmon, domestication, gene flow, single nucleotide polymorphisms

## Abstract

Genetic introgression of domesticated to wild conspecifics is of great concern to the genetic integrity and viability of the wild populations. Therefore, we need tools that can be used for monitoring unidirectional gene flow from domesticated to wild populations. A challenge to quantitation of unidirectional gene flow is that both the donor and the recipient population may be genetically substructured and that the subpopulations are subjected to genetic drift and may exchange migrants between one another. We develop a standardized method for quantifying and monitoring domesticated to wild gene flow and demonstrate its usefulness to farm and wild Atlantic salmon as a model species. The challenge of having several wild and farm populations was circumvented by in silico generating one analytical center point for farm and wild salmon, respectively. Distributions for the probability that an individual is wild were generated from individual-based analyses of observed wild and farm genotypes using STRUCTURE. We show that estimates of proportions of the genome being of domesticated origin in a particular wild population can be obtained without having a historical reference sample for the same population. The main advantages of the method presented are the standardized way in which genetic processes within and between populations are taken into account, and the individual-based analyses giving estimates for each individual independent of other individuals. The method makes use of established software, and as long as genetic markers showing generic genetic differences between domesticated and wild populations are available, it can be applied to all species with unidirectional gene flow. Results from our method are easy to interpret and understand, and will serve as a powerful tool for management, especially because there is no need for a specific historical wild reference sample.

## Introduction

Large-scale releases of plants and animals have the potential to augment population sizes, but also to cause adverse genetic changes in wild populations such as loss of genetic variation, loss of adaptation and change of population composition and structure (Ryman et al. 1995; Laikre et al. 2010). Populations of many species are being kept in captivity and subsequently deliberately or accidentally released in nature. Domesticated populations become genetically adapted to the captive environment rather than the natural environment. In commercial breeding programs, populations are also subjected to directional artificial selection for commercially important traits. The effect of releases of specimens held in captivity on wild populations depends on the number of generations in captivity, selection intensity, genetic variation, and the level of genetic introgression to the wild gene pool (Lynch and O'Hely 2001; Frankham 2008). Genetic introgression from domesticated to wild populations occurs at large scales in plants (Ellstrand et al. 1999), mammals (Randi and Lucchini 2002; Frantz et al. 2013; Feulner et al. 2013), and fishes (Goldburg and Naylor 2005). In 1996, FAO listed 103 fish species used in aquaculture worldwide (Garibaldi 1996). Because most of these species are being cultured in areas with wild conspecifics, there is a growing concern for negative effects on wild population from interbreeding with cultured fish (Svåsand et al. 2007 and references therein). Adverse genetic changes, following releases of cultured individuals, are recognized and documented in fish populations (Araki and Schmid 2010), but are rarely monitored – often because of a limited availability of molecular genetic markers and statistical methods to monitor genetic changes caused by releases of conspecific populations.

Atlantic salmon is suitable as a model for how to monitor genetic impact of domesticated populations on their wild conspecific. The reasons for this are the recent and well documented history of salmon farming, a documented lower fitness in farmed salmon compared to wild salmon, the known population structure of wild populations, large proportions of escaped farm salmon in wild populations, the development of molecular genetic tools, and documented genetic introgression.

A successful Atlantic salmon aquaculture industry now produces *c*. 1000 times more farm salmon than the total catches of wild Atlantic salmon worldwide (ICES 2012). Even though a small fraction of the farm salmon escapes from captivity, they can make up a large proportion of the spawning population in many rivers, outnumbering wild spawners in some of them (Fiske et al. 2006). Farm Atlantic salmon differ genetically from wild salmon for several reasons: (1) they originate from a small number of spawners from a limited number of wild (Norwegian) salmon populations (Gjedrem et al. 1991; Gjøen and Bentsen 1997), now being farmed over large areas; (2) they have undergone artificial selection for increased growth rate, reduced early sexual maturation, increased resistance to selected disease agents, and for various flesh parameters (Gjedrem and Baranski 2009); and (3) they are being adapted to the captive environment by natural selection. Recently, it was shown, by analyzing a large number of single nucleotide polymorphisms (SNPs), that it is possible to distinguish farm Atlantic salmon from wild Atlantic salmon using a set of SNPs that collectively are diagnostic for the two groups, irrespective of population of origin (Karlsson et al. 2011). Genetic drift operates on any finite population, and although a population may consist of hundreds of millions of individuals in farm production, the effective population size of the breeding kernels may be quite small (Karlsson et al. 2010). As long as the farm populations are genetically different and do not cluster as a group, different from the wild populations, it is a formidable task to disentangle the sequence of introgression between one or more groups of farm escaped salmon and the wild population (Besnier et al. 2011). The set of genetic markers (SNPs) identified by Karlsson et al. (2011) enable identification of generic genetic differences between wild and farm salmon. Nevertheless, the substructuring between populations remains. This substructure makes it a challenge to quantitate accumulated farm to wild gene flow because it is not possible to disentangle or keep track of the relative impact from all possible farm populations on a wild population. At the same time, wild populations exchange migrants naturally on a limited, but variable, scale and are also of limited size, sometimes featuring only a few tens of individuals in the spawning population. Our approach to circumvent these problems is to make two in silico center points (farm and wild) to which single individuals are probabilistically assigned. To quantitate genetic introgression, estimates of probabilities of belonging to the in silico center points obtained from admixed populations are compared to estimates from nonadmixed reference samples of wild salmon and farm salmon from the breeding kernels, respectively.

In other studies, all samples of interest are commonly being analyzed collectively in STRUCTURE. However, STRUCTURE is sensitive for differences in sample size, in particular when a group of samples are genetically heterogeneous (Kalinowski 2011). Consequently, if a whole group of individuals are analyzed simultaneously instead of being analyzed one by one, the estimated probabilities of belonging to an a priori assumed number of populations will be biased.

Our main objective was to develop a standardized method for estimating changes in the genetic composition of a recipient (wild) population, caused by gene flow from a diverse source of (farm) populations that have some genetic characteristics in common. Specifically, we wanted to develop a method for (1) estimating genetic change resulting from unidirectional gene flow when genetic drift and gene flow from other wild populations are also affecting the gene pool; (2) estimating farm to wild genetic introgression in a wild population without the need of a historical reference sample from the same population; and (3) estimating the probability of single individuals being of farm or wild origin. We develop the method using Atlantic salmon as a model, but emphasize that the approach is a general one provided the necessary molecular genetic markers are at hand.

## Material and Methods

Our method consists of the following steps: (1) identify a set of reference populations defining two genetically distinct groups; (2) generate a center point for each of these genetic groups; (3) genetic assignment of the reference individuals to the center points to generate a reference probability distribution for belonging to one of the two center points; (4) genetic assignment of admixed individuals to the center points to generate an admixed probability distribution; and (5) the admixed and the reference probability distributions are compared to quantitate genetic introgression.

### Reference populations

The farm and wild reference populations (Table S1) are largely the same as those used by Karlsson et al. (2011) except that a new breeding strain from AquaGen (AG -08) is included, more historical wild populations are represented in this study (20 instead of 13), and the northeasternmost region of Norway is excluded. The AG -08 sample from AquaGen represents a new strain created by pooling the previous four isolated strains into one large breeding kernel. Northeasternmost Norway represents a different phylogeographic grouping than the rest of wild Atlantic salmon in Norway (Bourret et al. 2013), and therefore needs to be treated separately. SNP genotyping was carried out by Centre for Integrative Genetics (CIGENE), Norwegian University of Life Sciences, Ås, using a Sequenom platform. Reliable genotypes were obtained from 59 SNPs described as being collectively diagnostic in differentiating between wild and farm salmon (Karlsson et al. 2011; Jensen et al. 2013). The genetic clustering of wild and farm reference populations was visualized in a principal coordinate analysis plot based on pairwise *F*_ST_ estimates, as implemented in GENALEX 6.0 (Peakall and Smouse 2006).

### Reference center points

To standardize the analysis, we created center points for the wild and farm populations based on the observed genotypes of the wild and farm reference samples. The rationale for creating these center points, instead of using many reference populations, is to standardize the way in which estimates of probability for belonging to the wild or farm cluster are obtained, and thus make all estimates comparable regardless of population of origin. For the genetic assignment, it is important that the center points represent populations in Hardy–Weinberg equilibrium, instead of being a heterogeneous group of individuals from genetically different populations (Kalinowski 2011). Consequently, the center points were created separately from wild and farm reference samples (Table S1). This was performed by randomly sampling, without replacement, an equal number from each population (based on the smallest samples size), 18 from the wild populations and 19 from the farm populations. Random mating within the wild and within the farm pool was allowed to restore Hardy–Weinberg equilibrium, using HybridLab (Nielsen et al. 2006). In this program, alleles are randomly picked conditionally on calculated allele frequencies at each locus from the parental genotype file(s). Each wild and farm in silico generated population constituted 100 individuals representing the center points for wild and farm salmon. Because the in silico generated center points were created based on the allele frequencies in the pooled groups, and not on observed genotypes, a sample size of 100 offspring is sufficiently large to represent the parental allele frequencies, and at the same time sufficiently small for conducting computationally demanding analyses in STRUCTURE (as described below).

### Assignment of reference individuals to center points

Assignment of wild and farm samples with real (observed) genotypic data to the wild and farm center points was conducted using STRUCTURE (Pritchard et al. 2000). We applied 50 000 repetitions as burn-in and 100 000 repetitions after burn-in, no a priori information of sampling locality, and assumed two populations (wild and farm). In STRUCTURE, individuals are probabilistically assigned to an a priori assumed number of populations based on their multi-locus genotype, so that deviations from Hardy–Weinberg and linkage equilibrium are minimized. To avoid biased results in STRUCTURE, as explained above, we analyzed one by one individual together with the center points, instead of all individuals of interest collectively, assuming two populations (*K* = 2). From the genetic assignment of reference individuals to the center points, we obtained a probability of belonging to the wild center point *P*(wild) for each individual. The corresponding probability of belonging to the farm center point is 1 − *P*(wild). Based on the individual *P*(wild) estimates for the reference samples, we generated probability distributions for wild and farm salmon, respectively. These probability distributions were then used for statistical testing if there had been a farm to wild gene flow, and for estimating proportions of wild genome left in admixed populations.

### Assignment of admixed individuals to center points

Estimates of *P*(wild) for admixed individuals are obtained in the exact same way as explained for reference individuals. From individual estimates of *P*(wild), we obtain a probability distribution for the admixed population.

### Quantitate genetic introgression

When evaluating a contemporary sample from a population, we are interested in testing whether or not the population has experienced genetic introgression. The null hypothesis is that the contemporary sample has the same mean *P*(wild) as the historical reference, and the alternative hypothesis is that the contemporary sample is admixed and has a lower mean *P*(wild). Before performing the analysis, the probabilities *P*(wild) are logit-transformed (Warton and Hui 2011). This test corresponds to a standard two sample test for comparing means. Under this null hypothesis, the two samples are from the same population so we can assume that they have equal variances. Arguing that all populations have the same variance for *P*(wild), this variance can be estimated from all the historical samples.

If we have a historical reference sample from a population, the two samples (historical and contemporary) can be compared directly by the test on two means, alternatively by calculating a confidence interval for the difference between the two means. When we evaluate the genetic introgression from escaped farm salmon into a wild salmon population *without* a historical reference sample, the test, or confidence interval estimation, will have an additional variance component caused by the variability in mean *P*(wild) value between wild populations. The variance in wild population mean *P*(wild) values can be estimated from the available wild references, assuming that the available wild references can be considered a random sample from all wild populations.

Because wild reference populations have an average *P*(wild) less than one, and farm reference populations have an average *P*(wild) above zero, we need to calibrate the scale accordingly. Hence, the proportion of wild genome left in an admixed population, was calculated as:


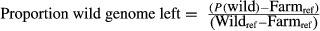


where 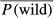 is the average *P*(wild) estimate for the admixed population, Farm_ref_ is the average *P*(wild) for the farm reference samples, and Wild_ref_ is the average *P*(wild) for the wild reference samples.

The power of identifying first-generation hybrids between farm and wild salmon was explored by simulating mating between individuals of wild and farm origin using observed genotype data. From wild individual genotypes, one allele was randomly sampled from each locus, generating one gamete that was merged with a correspondingly generated gamete from farm individuals. Five randomly generated first-generation hybrids were selected from each pair of wild and farm populations (260 pairs × 5 = 1300). Hybrid simulations were conducted using a script written in R (R Development Core Team 2013). Each generated first-generation hybrid was analyzed together with the wild and farm center points, using STRUCTURE as described above.

To further explore the power of the method to quantitate farm to wild gene flow, offspring from experimental crossings between farm and wild salmon were analyzed. Crossings were conducted between salmon from AquaGen farm strain and River Namsen at the NINA Research Station at Ims, Norway. The following crossings were made, corresponding to all possible admixed and nonadmixed groups after two generations of interbreeding between wild and farm salmon: The first generation including pure wild, pure farm, first-generation hybrids (H), and in the second generation also backcrosses of first-generation hybrids to wild (BCW) and farm (BCF), and crosses between first-generation hybrids (2GH). Thirty individuals from each group were analyzed using STRUCTURE as described above. This material enabled us to investigate a realistic outcome from two generations of interbreeding between escaped farm salmon and wild salmon, assuming 20% farm escaped salmon participating in each breeding event, and applying relative fitness components as described by Hindar et al. (2006).

## Results

**Figure 1:**
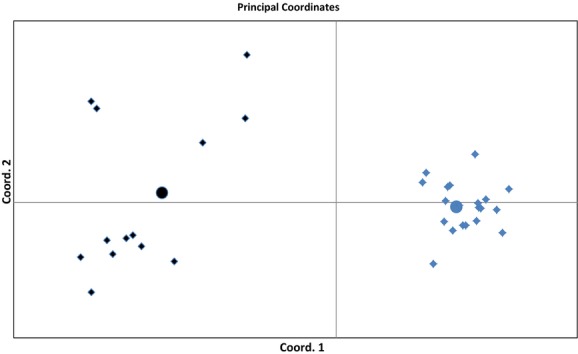
PCoA plot from pairwise *F*_ST_ estimates between farm (black diamonds), wild (blue diamonds) and in silico generated farm (black circle) and wild (blue circle) center points. The first and the second axis explained 55% and 16% of the variation, respectively.

The generated wild and farm center points were located in the center of the wild and farm reference population clusters, respectively (Fig. 1). These two center points, containing 100 individual each were subsequently used in STRUCTURE for the analyses of wild and farm salmon.

**Figure 2:**
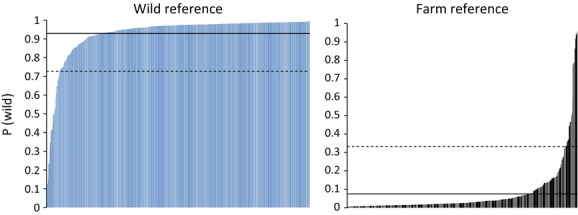
Distribution of probabilities for belonging to one (wild) of two assumed populations (wild and farm) [*P*(wild)] using STRUCTURE for historical wild reference samples (blue bars) and farm references (black bars). Estimates were obtained from analyzing individuals one by one together with wild and farm in silico generated center points. Solid horizontal line is average, and dashed line is lower 5 percentile and upper 95 percentile for wild and farm reference samples, respectively.

The estimated probabilities for the reference wild and farm populations of belonging to the wild center point *P*(wild) serve as reference distributions for defining wild and farm salmon, respectively (Fig. 2). The wild salmon had an average *P*(wild) estimate of 0.93 with a lower 5 percentile of 0.73, while corresponding estimates for the farm salmon was 0.07 with an upper 95 percentile of 0.33. Population-specific mean estimates of *P*(wild) varied between 0.88 (Orkla -84) and 0.98 (Ferga -91) for the wild populations, and between 0.018 (AG -01) and 0.18 (AG -99) for the farm populations.

**Figure 3:**
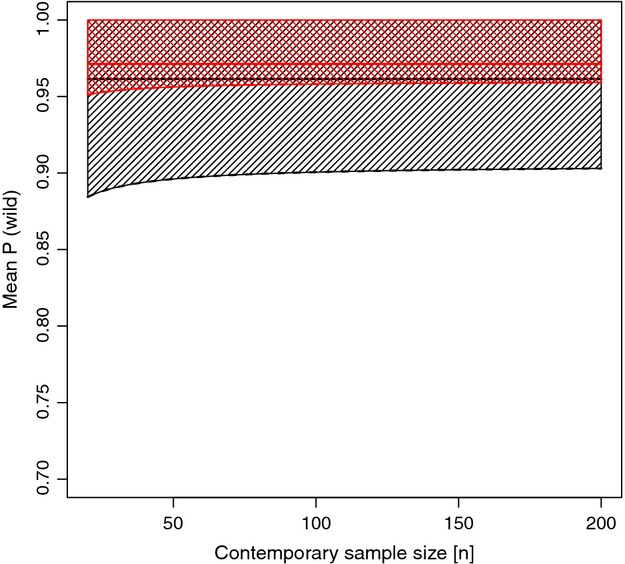
Acceptance region (shaded area) and critical value (lower, dashed line) for a hypothesis test comparing means from a historical reference sample and a contemporary sample, shown as a function of the contemporary sample size. The alternative hypothesis states that the contemporary sample mean is smaller than the historical reference mean. Black shaded area and line indicates the test without a population-specific historical reference, whereas red shaded area and line the test for one of our populations with a historical reference sample. The straight, solid lines give the means of the wild references.

To evaluate whether or not a wild population is significantly affected by genetic introgression of farm salmon, *P*(wild) values from a contemporary sample can be compared to: (1) the distribution from the historical reference sample from the same population, if this exists; or (2) the distribution of values from all wild reference samples, assuming that the historical reference populations we have can be considered a random sample from all populations. Figure 3 illustrates the acceptance regions (shaded areas) and critical values (lower, dashed lines) for the contemporary mean as a function of the size of the contemporary sample, with black area and line indicating the test without a specific historical reference, and red illustrating the critical value for one of our wild populations with a specific historical reference sample (*n* = 33). If the observed mean *P*(wild) from the contemporary sample is below the lower dashed line, we can reject the null hypothesis of no change in mean *P*(wild), at a 0.05 significance level.

**Figure 4:**
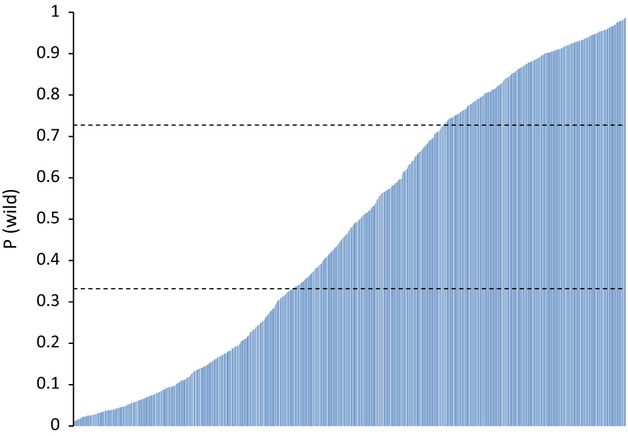
Distribution of probabilities for belonging to one of two assumed populations (wild and farm) [*P*(wild)] using STRUCTURE for in silico generated crossing (hybrids) between wild and farm salmon reference samples (Table S1) using real genotype data. Estimates were obtained from analyzing individuals one by one together with wild and farm in silico generated center points. Upper and lower horizontal dashed line is the lower 5 percentile and the upper 95 percentile from the probability distribution from wild and farm references.

Estimates of *P*(wild) for 1300 simulated first-generation hybrids between wild and farm salmon ranged from 0.01 to 0.99, with an average of 0.48 (Fig. 4). Hence, hybrid individuals cover estimates typical for both pure wild and pure farm salmon, and identification of first-generation hybrids is therefore expected to be uncertain at the individual level. Nevertheless, 27% of the hybrids had *P*(wild) estimates between the lower 5 percentile for wild salmon (0.73) and the upper 95 percentile for farm salmon (0.33), and these can therefore be classified as admixed. Furthermore, the average estimate of *P*(wild) was 0.48 which in relation to the average estimate of wild salmon (0.93) and the farm salmon (0.07) corresponds to a proportion of wild genome left of 47.7%, which is close to the expected 50%.

Offspring from experimental crossing between wild and farm salmon were also analyzed, with all possible admixed groups that can be generated after two generations of interbreeding with escaped farm salmon. A realistic scenario of genetic introgression from escaped farm salmon was simulated for two generations of 20% escaped farm salmon in the spawning population, whereby expected proportions of the pure wild (0.706), hybrid groups (H = 0.107, BCW = 0.167, BCF = 0.007, 2GH = 0.009), and pure farm (0.004) salmon was obtained with an overall expected proportion of wild genome left of 89%. From resampling (1000 times) of individuals with estimates of *P*(wild) from these groups, conditional on expected proportions, the estimated average proportion of wild genome left was 87%, that is, close to the expectation. The average estimate was the same from a sample size of 30 compared to a sample size of 100, but with a broader confidence interval from a sample of 30 individuals (2.5% lower = 74%, 97.5% upper = 97%) compared with a sample size of 100 (79–93%).

## Discussion

We have developed a standardized method for estimating unidirectional introgression, using Atlantic salmon as a model organism. In doing this, we wanted to utilize the power of the recently developed set of SNP markers that collectively differentiate between wild and farm salmon irrespective of the population of origin (Karlsson et al. 2011).

The main challenge when trying to quantitate farm to wild gene flow is to isolate its effects from those of genetic drift and gene flow from other wild populations. Analyses of temporal genetic change in Norwegian salmon populations were recently carried out by Glover et al. (2012, 2013). In the latter study, they used approximate Bayesian computation to quantitate the amount of gene flow from farm salmon needed to explain an observed temporal genetic change in a wild population. The underlying analyses in the studies of Glover et al. (2012, 2013) were done at the population level, with population-specific estimates and assumptions for effective population size and migration rates from either a nearby wild population or from farm salmon. Here, we suggest an alternative approach where the underlying analyses are done at the individual level. From analyzing a large set of historical wild salmon and salmon from the dominating breeding kernels, we were able to define an expected distribution of estimates for pure wild salmon and for pure farm salmon with a high discrimination, also at the individual level. Because analyses are done at the individual level, the obtained probability distribution includes all evolutionary processes that act on the genetic composition of the individual, including genetic drift and gene flow between wild populations.

Our approach enables quantitation of farm genetic introgression from a contemporary sample without having historical samples from this particular population. Historical reference samples are expected to increase the precision if the historical samples constitute a good representation of the spawning population before any impact of escaped farm salmon. The distribution of individual estimates makes it possible to evaluate the underlying events. For example, the same mean estimate of farm introgression in the population can either result from a few individuals of pure farm origin or from a larger number of admixed individuals.

As there are many genetically differentiated farm strains in Norway, introgression is expected to occur in a complicated way, involving escaped farm salmon of different origin in a spawning population. It is impossible to track, or reconstruct, the origin of farm salmon involved in interbreeding with wild salmon, especially across several generations. For this reason, we generated an analytical center point for farm salmon and one for wild salmon. STRUCTURE was then used to estimate, for each individual, the probability of belonging to one of these two center points. It is important to note that the center points were only used for STRUCTURE analyses and they did not themselves define the wild and farm reference probability distributions. The most important method assumption is that the samples of historical wild salmon and samples of farm salmon are actually representing wild and farm salmon, and can be used as references for quantifying introgression of farm salmon. In the absence of historical reference samples from a particular population, all available historical wild samples may serve as references. Estimate of proportion of wild genome left in a contemporary sample may then be obtained using the average *P*(wild) estimates from all historical samples, and/or from the historical populations with the highest and lowest *P*(wild) estimates, to give a possible range of the level of genetic introgression. To evaluate the significance of the estimates, the lower 5 percentile of expected estimates, conditional on sample size, can be used. The lower 5 percentile can either be generated from the overall sample reference distribution (Fig. 3), or from historical reference samples from specific populations.

We tested the precision of the method by analyzing in silico generated first-generation hybrids between wild and farm salmon, and by analyzing offspring from experimental crossing between farm and wild salmon. Results from these analyses convincingly showed that the method has a high precision in quantifying genetic introgression of farm salmon at the population level. First-generation hybrids gave a large range of estimates of *P*(wild). One third of the first-generation hybrids had intermediate *P*(wild) estimates and were unlikely to be of either pure farm or pure wild origin. In an admixed population containing two or more generations of hybrids and backcrosses, it will become increasingly difficult to classify single individuals as pure wild or pure farm.

The method presented here is generic and applicable to all species for which there is a set of genetic markers that can differentiate between wild and domesticated individuals irrespectively of population of origin. The complexity and/or possibilities for finding generic genetic differences is expected to vary between species and depends on the wild origin of the domesticated populations, number of different breeding populations, number of generations in captivity, effective population size (genetic drift), strength of artificial selection, etc. In addition, farm salmon, and other domesticated species, are constantly changing genetically from directional selection and genetic drift, and new farm populations may be constructed. This makes tracing of domesticated individuals a moving target, which may require updates of samples of the domesticated populations. At the same time, the available genetic tools are expected to improve, both in terms of genetic markers and analytical methods. In order to make the transition between the development of new genetic markers and the application of these as effective as possible, it is important to have a dynamic analytical platform, allowing for new samples to be included and new markers to be applied. We believe the method presented here will serve this purpose.

## Supporting Information

**Table S1.** Wild populations (a) and farm breeding strains (b) of Atlantic salmon and corresponding sample sizes (*N*) genotyped for 59 SNPs. Numbers after population names are sampling years.
