# Supplementary material for: A standardized method for quantifying unidirectional genetic introgression
Source: Ecol Evol. 2014 Jul 28;4(16):3256–63. doi: 10.1002/ece3.1169 (PMC4222212; doi:10.1002/ece3.1169)
Supplement: Supplementary file 1 — Table S1. Wild populations (a) and farm breeding strains (b) of Atlantic salmon and corresponding sample sizes (N) genotyped for 59 SNPs. Numbers after population names are sampling years. [file ece30004-3256-sd1.docx]

Table S1. Wild populations (a) and farmed breeding strains (b) of Atlantic salmon and corresponding sample sizes (N) genotyped for 59 SNPs. Numbers after population names are sampling years

| Populations | N |
| --- | --- |
| (a) |  |
| Salangselva -89, -90 | 61 |
| Saltdalselva -77, -78 | 27 |
| Namsen -78 | 52 |
| Ferga -91 | 24 |
| Gaula -89, -90 | 39 |
| Orkla -84 | 22 |
| Surna -77, -78 | 45 |
| Driva -77 | 18 |
| Eira -89 | 59 |
| Rauma -74, -76, -77, -79, -83 | 33 |
| Måna -86, -90, -91 | 34 |
| Gloppenelva -89 | 26 |
| Lærdalselva -77, -78 | 47 |
| Vosso -77, -78 | 40 |
| Granvin -89 | 33 |
| Oselva -51, -52, -53 | 31 |
| Etne -89 | 48 |
| Suldalslågen -79, -80 | 50 |
| Figgjo -89 | 47 |
| Numedalslågen -89 | 50 |
| Total | 786 |
| (b) |  |
| AG -98 | 27 |
| AG -99 | 35 |
| AG -00 | 33 |
| AG -01 | 50 |
| AG -08 | 95 |
| Mowi -04 | 19 |
| Mowi -05 | 19 |
| Mowi -08 | 20 |
| Mowi -09 | 20 |
| SB -04 | 46 |
| SB -05 | 47 |
| SB -06 | 44 |
| SB -07 | 48 |
| Total | 503 |
